# Inside epoxyeicosatrienoic acids and cardiovascular disease

**DOI:** 10.3389/fphar.2014.00239

**Published:** 2014-11-10

**Authors:** Stefania Tacconelli, Paola Patrignani

**Affiliations:** Department of Neuroscience, Imaging and Clinical Science, Center of Excellence on Aging (CeSI), “Gabriele d’Annunzio” UniversityChieti, Italy

**Keywords:** hypertension, epoxyeicosatrienoic acids, cytochrome P450, epoxygenases, soluble epoxide hydrolase

## Abstract

Epoxyeicosatrienoic acids (EETs) generated from arachidonic acid through cytochrome P450 (CYP) epoxygenases have many biological functions. Importantly, CYP epoxygenase-derived EETs are involved in the maintenance of cardiovascular homeostasis. In fact, in addition to their potent vasodilating effect, EETs have potent anti-inflammatory properties, inhibit platelet aggregation, promote fibrinolysis, and reduce vascular smooth muscle cell proliferation. All EETs are metabolized to the less active dihydroxyeicosatrienoic acids by soluble epoxide hydrolase (sEH). Numerous evidences support the role of altered EET biosynthesis in the pathophysiology of hypertension and suggest the utility of antihypertensive strategies that increase CYP-derived EET or EET analogs. Indeed, a number of studies have demonstrated that EET analogs and sEH inhibitors induce vasodilation, lower blood pressure and decrease inflammation. Some of these agents are currently under evaluation in clinical trials for treatment of hypertension and diabetes. However, the role of CYP epoxygenases and of the metabolites generated in cancer progression may limit the use of these drugs in humans.

## EPOXYEICOSATRIENOIC ACIDS: SYNTHESIS AND METABOLISM

**FIGURE 1:**
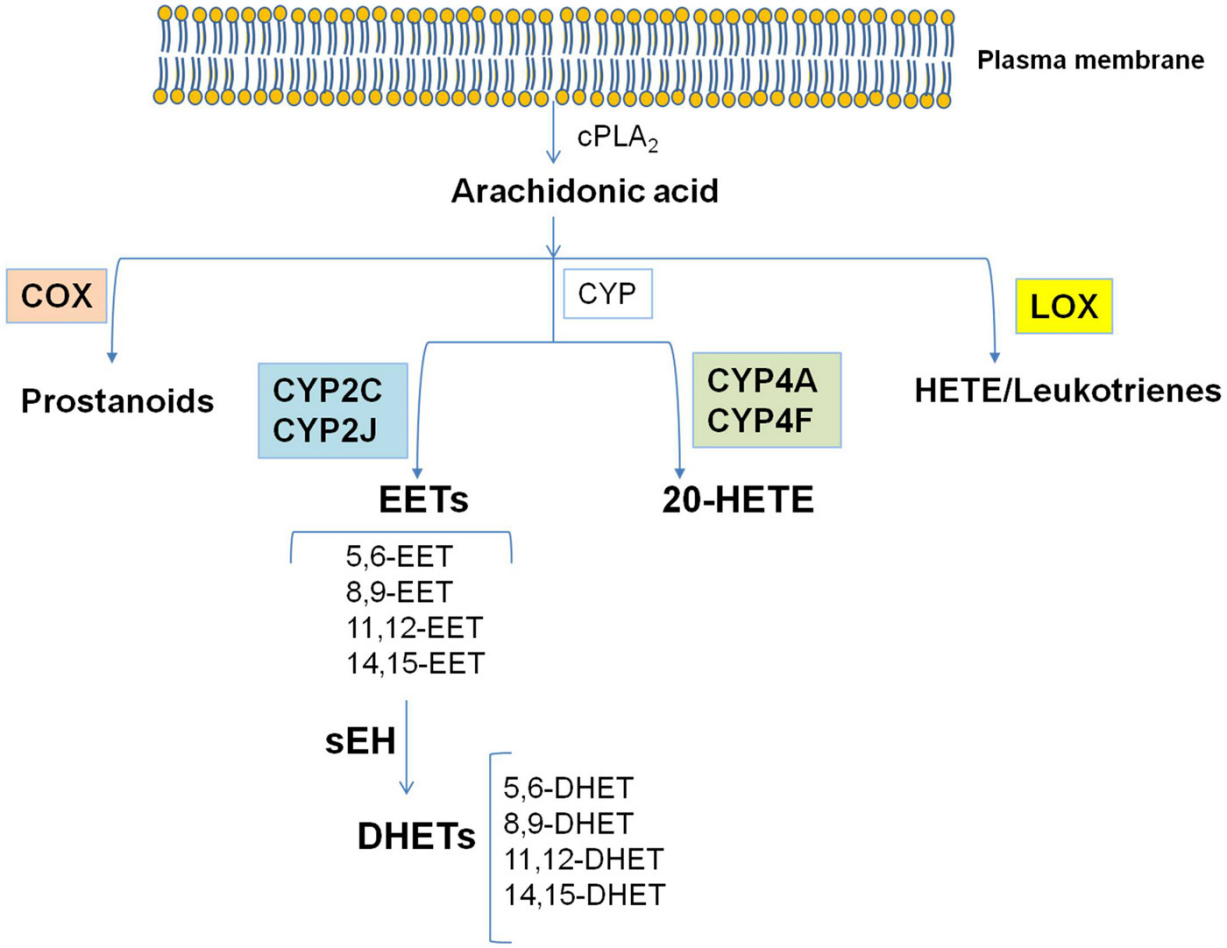
**The cascade of arachidonic acid (AA).** AA is a polyunsaturated omega-6 fatty acid which is released from the sn2 position of membrane phospholipids by the activity of cPLA_2_. Free AA can be metabolized to eicosanoids through three major pathways: the cyclooxygenase (COX) pathway, the lipoxygenase (LOX) pathway, and the cytochrome P450 (CYP) pathway. In the CYP pathway, AA is converted to epoxyeicosatrienoic acids (EETs) and 20-HETE by CYP epoxygenases and CYP ω-hydroxylases, respectively. All EETs are then further metabolized by soluble epoxide hydrolase (sEH) forming the less active dihydroxyeicosatrienoic acids (DHETs). Modified from Imig and Hammock (2009).

Arachidonic acid (AA) is a polyunsaturated omega-6 fatty acid which is released from the sn2 position of membrane phospholipids by the activity of phospholipases (PLs) and among them it is noteworthy the role of cytosolic(c)PLA_2_. Free AA can be metabolized to eicosanoids through three major pathways: (i) the cyclooxygenase (COX) pathway, which generates prostanoids; (ii) the lipoxygenase (LOX) pathway, which generates leukotrienes and hydroxyeicosatetraenoic acids (HETEs); (iii) the cytochrome P450 (CYP) pathway, which includes CYP epoxygenase and CYP ω-hydroxylase enzymes (Wang and Dubois, 2012; Oni-Orisan et al., 2014). CYP epoxygenases, such as members of the CYP2C and CYP2J families, metabolize AA to four biologically active epoxyeicosatrienoic acids (EETs; 5, 6-EET, 8, 9-EET, 11, 12-EET, and 14, 15-EET; **Figure 1**). Among the members of the CYP2C and CYP2J families of CYP enzymes, CYP2J2, CYP2C8, and CYP2C9 are the predominant epoxygenase isoforms that convert AA into EETs. CYP ω-hydroxylases convert AA to HETEs. CYP4A and CYP4F enzymes mainly catalyze the ω-hydroxylation of AA to 20-HETE (Powell et al., 1998; **Figure 1**). In addition, CYP1A1, CYP1B1, and CYP2E1 were reported to catalyze the formation of different regioisomers of HETEs.

EETs are synthesized by cells which express CYP epoxygenase activity. The CYP epoxygenase inserts an oxygen atom on a carbon attached to one of the double bonds of AA, and the double bond is reduced as the epoxide forms (Spector, 2009). Each CYP epoxygenase produces several regioisomers, with one form usually predominating. Each regioisomer contains two R/S enantiomeric forms in different proportions (Spector, 2009). Because the regioisomers have a number of similar metabolic and functional properties, all the EETs can be generally considered as a single class of compounds, even if some qualitative differences in the actions of the various regioisomers exist (Spector, 2009). All EETs are then further metabolized by soluble epoxide hydrolase (sEH, EC 3.3.2.7–11; Wang and Dubois, 2012; Bellien and Joannides, 2013), which acts by opening epoxides to diols by the addition of water, forming the less active dihydroxyeicosatrienoic acids (DHETs). This conversion of EET to DHET by sEH attenuates most biological effects of EETs, making sEH a target for increasing and prolonging the actions of EETs (Spector, 2009). In addition, EETs undergo β-oxidation, forming 16-carbon epoxy-derivatives that accumulate in the extracellular fluid, and they can be chain-elongated to form 22-carbon derivatives that are incorporated into phospholipids. EETs are also readily incorporated into cellular membranes via esterification to phospholipids for subsequent release by phospholipases (Spector, 2009). Endothelial cells are a major site of EET incorporation, primarily acylated at the sn2 position of phospholipids and capable of being released by PLA_2_ (Weintraub et al., 1999).

## BIOLOGICAL EFFECTS OF EETs

Epoxygenase enzymes are localized in endothelial and vascular smooth muscle cells, and also in astrocytes and cardiomyocytes (Imig, 2012; Wang and Dubois, 2012; Oni-Orisan et al., 2014). While 20-HETE is a vasoconstrictor, EETs are vasodilators, except in the pulmonary bed (Bellien and Joannides, 2013). The mechanisms of EET-induced vasodilatation are complex, but increasing evidences have suggested the involvement of endothelial and smooth muscle membrane receptors (Bellien et al., 2011; Chen et al., 2011). In fact, EETs are able to diffuse from the endothelial cells to activate large conductance calcium-activated potassium (BKCa) channels located on the smooth muscle cells, causing their hyperpolarization and relaxation. Then, the endothelial hyperpolarization is transmitted to the smooth muscle through the gap junctions, or the accumulation of K^+^ released from the endothelial KCa channels into the myoendothelial space induces smooth muscle hyperpolarization by the activation of inward rectifying potassium channels and/or Na^+^/K^+^-ATPase (Archer et al., 2003; Huang et al., 2005). The potent vasodilatory effects of EETs are more pronounced in the presence of inhibition of prostacyclin and nitric oxide biosynthesis (Deng et al., 2010). For all of these reasons, CYP-derived EETs are considered as one of the primary endothelium-derived hyperpolarizing factors (EDHFs; Bellien and Joannides, 2013).

**FIGURE 2:**
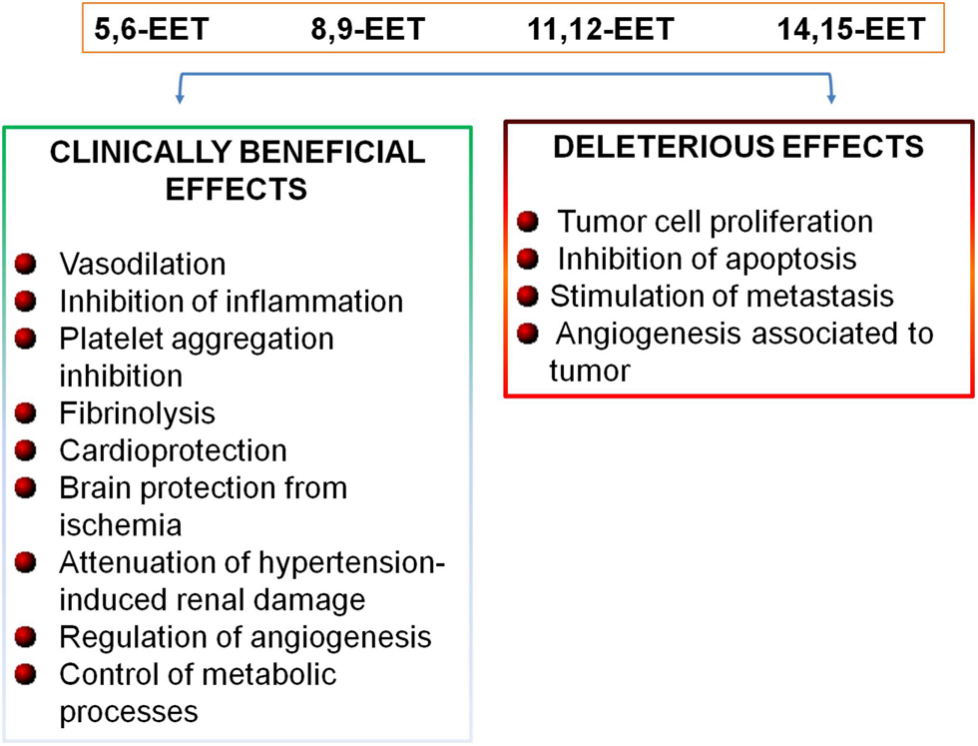
**Biological effects of EETs**.

Recently, in addition to maintain vascular tone, it has been established a further role of CYP epoxygenase-derived EETs in the maintenance of cardiovascular homeostasis (**Figure 2**). In fact, EETs induce vasodilation and exert anti-inflammatory effects in blood vessels in an autocrine manner (Fleming, 2008), limiting leukocyte adhesion and transmigration across the endothelium, inhibiting platelet aggregation, promoting fibrinolysis, and reducing vascular smooth muscle cell proliferation (**Figure 2**; Fleming, 2008; Deng et al., 2010; Bellien and Joannides, 2013). EETs can protect the myocardium and brain from ischemia, attenuate hypertension-induced renal damage, and reduce cigarette smoke-induced lung inflammation (**Figure 2**; Imig and Hammock, 2009; Deng et al., 2010; Wang and Dubois, 2012). EETs control these different functions by inducing endothelial cell proliferation, survival, and stimulating renal epithelial cell proliferation and survival through multiple signaling pathways (Xu et al., 2011). EETs play a role in the regulation of intracellular Ca^2+^ levels and endoplasmic reticulum homeostasis, through the expression of the sarcoplasmic/endoplasmic reticulum calcium ATPase (SERCA2a) which transfers Ca^2+^ from the cytosol of the cardiomyocyte to the lumen of the sarcoplasmic reticulum during muscle relaxation (Wang et al., 2014).

In addition, EETs play important roles in the regulation of angiogenesis and in the control of metabolic processes (**Figure 2**; Fleming, 2007; Lorthioir et al., 2012). Moreover, recent emerging evidences have indicated that EETs are involved in cancer biology; in fact, it has been shown that EETs are critical for primary tumor growth and metastasis, by directly promoting cancer cell proliferation, survival, migration and invasion (**Figure 2**; Jiang et al., 2007; Panigrahy et al., 2012; Wang and Dubois, 2012).

## EETs IN CARDIOVASCULAR DISEASE

### HYPERTENSION

Many evidences suggest that alteration in EET pathway contribute to the pathophysiology of hypertension, including blood pressure elevation, endothelial dysfunction and end-organ damage. The infusion of angiotensin II (Ang II), a potent vessel constrictor, elevates blood pressure in various animal models (Imig et al., 2002). It has been established that Ang II stimulates 20-HETE synthesis in renal microvessels and decreases EET levels by downregulating epoxygenases and increasing their degradation by increasing expression and activity of sEH (Croft et al., 2000; Alonso-Galicia et al., 2002; Ai et al., 2007). In addition, numerous animal studies have established the role EETs in blood pressure regulation. Mice lacking the sEH gene (epoxide hydrolase 2, Ephx2^-/-^) have significantly higher circulating EET levels and lower blood pressure when compared with wild-type mice. Renal production of DHETs was decreased and EET formation increased in the Ephx2^-/-^mice, suggesting an important role for epoxygenase metabolism in the regulation of blood pressure (Sinal et al., 2000). In addition, the administration of a sEH inhibitor (sEHI) significantly lowers blood pressure in various rodent models of hypertension (Sinal et al., 2000; Manhiani et al., 2009). The administration of a single dose of a sEH inhibitor [sEHI; *N,N*′-dicyclohexyl urea (DCU)] to Ang II-infused rats greatly increased the level of EETs, decreased urinary DHET excretion, and lowered systolic blood pressure, thus reversing the hypertensive phenotype typical of the spontaneously hypertensive rats (Yu et al., 2000; Imig et al., 2002). Moreover, reduced myogenic constriction in the arterioles of Ephx2^-/-^ mice has been recently found (Sun et al., 2014). This effect was mediated by increased endothelial EET bioavailability which potentiates vasodilator responses that counteract pressure-induced vasoconstriction to lower blood pressure.

The central role of renal sEH in the development of hypertension is also sustained by the finding that kidneys of the spontaneously hypertensive rats have increased expression of sEH and urinary DHET excretion (Imig et al., 2002). Moreover, Kujal et al. (2014) have recently shown that renin (Ren)-2 transgenic rats (TGR) after 5/6 renal mass reduction (i.e., a model of chronic kidney disease associated with Ang II-dependent hypertension), exhibit a profound deficiency of intrarenal availability of EETs, that was mitigated by the use of a sEH inhibitor [*cis*-4-[4-(3-adamantan-1-yl-ureido)cyclohexyloxy]benzoic acid c-(AUCB)]. This phenomenon was associated with renoprotective actions.

Renovascular disease (RVD) represents a relatively rare form of secondary hypertension; it is associated with increased cardiovascular mortality and it is one of the prevalent causes of end-stage renal failure (Johansson et al., 1999; Fatica et al., 2001). In RVD, the activation of the renin-angiotensin system leads to increased Ang II activity which contributes to vasoconstriction, increased endothelin release, vascular remodeling, extracellular matrix deposition, and accelerated atherogenesis and glomerulosclerosis (Kim and Iwao, 2000). This disease is strictly associated with enhanced lipid peroxidation related to activation of the renin-angiotensin system (Minuz et al., 2002). In patients with RVD-associated hypertension, it has been found a decrease in EET plasma levels that supports a pivotal role of EETs in vascular homeostasis (Minuz et al., 2008). Interestingly, the ratio of plasma EETs:DHETs, which provides an index of the activity of sEH was reduced in patients with RVD or with essential hypertension compared to control subjects, suggesting the increase of sEH activity. In contrast to EETs, plasma 20-HETE levels were higher in patients with RVD compared to subjects with essential hypertension and healthy controls. In RVD, plasma 20-HETE significantly correlated with plasma renin activity, thus suggesting its role in the elevation of blood pressure through the possible increase of vasomotion and vascular reactivity (McGiff and Quilley, 1999). A significant reduction of the urinary excretion of 20-HETE was detected in RVD (Minuz et al., 2008). This finding may reflect a decrease in 20-HETE production by the thick ascending limb and proximal tubules which may lead to increased Na^+^ reabsorption in these tubular segments.

In pregnancy-induced hypertension, the urinary excretion of DHETs is increased versus healthy pregnant women which may implicate an increased degradation of EETs (Catella et al., 1990). Differently, Jiang et al. (2013) found a decreased urinary excretion of DHETs in preeclampsia which can be interpreted as evidence of deficient renal EET formation in preeclamptic pregnancy. However, direct comparison of the two studies is complicated by the severity of hypertension and clinical conditions of patients in the study by Catella et al. (1990; mean blood pressure 182/112 mmHg, reduced platelet count).

Plasma EET levels are higher in preeclamptic and normotensive pregnant women than in non-pregnant controls (Jiang et al., 2013). Furthermore, plasma levels of EETs are 3-fold higher in fetal than in maternal blood. Altogether these results suggest that the vasodilatatory and antiinflammatory activities of EETs may represent the basis for cellular protective actions in the fetoplacental unit and vascular homeostasis in pregnancy (Jiang et al., 2013).

Some genetic evidences in humans have supported the crucial role of the epoxygenase pathway in hypertension (Fava et al., 2012). In fact, it has been found that a single nucleotide polymorphism (SNP) in the CYP2J2 gene (the CYP2J2*7 genotype) was associated with hypertension in males, but not in females of a Caucasian population (King et al., 2005). It is known that the SNP CYP2J2*7 interferes with a binding site for the transcription factor Sp1 with consequent reduction in the plasma levels of EETs *in vivo* (King et al., 2002; Spiecker et al., 2004). In addition, CYP2C8, CYP2C9, and also EPHX2 genetic variants have been associated to myocardial infarction and cardiovascular disease (Spiecker et al., 2004; Marciante et al., 2008).

### CORONARY ARTERY DISEASE AND OTHER CARDIOVASCULAR RISK FACTORS

Increased EET plasma levels were observed in patients with stable angiographically confirmed coronary artery disease (CAD; Theken et al., 2012), without any modification in DHET level, suggesting that, in these particular conditions, an upregulation of CYP epoxygenase activity/expression may serve as a defensive mechanism.

In a population of patients with stable, angiographically confirmed CAD and healthy, Theken et al. (2012) aimed to identify clinical factors that influence CYP epoxygenase, sEH, and CYP ω-hydroxylase metabolism. Obesity was significantly associated with low plasma EET levels and 14,15-EET:14,15-DHET ratios (a biomarker of sEH metabolism). Age, diabetes, and cigarette smoking also were significantly associated with CYP epoxygenase and sEH metabolic activity, while only renin-angiotensin system inhibitor use was associated with CYP ω-hydroxylase metabolic activity. Compared to healthy volunteers, both obese and non-obese CAD patients had significantly higher plasma EETs and epoxide:diol ratios, whereas no difference in 20-HETE levels was observed (Theken et al., 2012). Collectively, these findings suggest that CYP-mediated eicosanoid metabolism is dysregulated in certain subsets of CAD patients, and demonstrate that biomarkers of CYP epoxygenase and sEH, but not CYP ω-hydroxylase, metabolism are altered in stable CAD patients relative to healthy individuals.

Recently, it was found a protective role of CYP2J2-derived EETs in heart failure (Wang et al., 2014). Thus CYP2J2-derived EETs may be a target for the development of drugs to prevent cardiac hypertrophy and cardiomyocyte apoptosis in heart failure.

## PHARMACOLOGICAL STRATEGIES TO MODULATE CYP-DERIVED EPOXYEICOSATRIENOIC ACIDS PATHWAY

The generation of transgenic mice with endothelial expression of the human CYP2J2 and CYP2C8 epoxygenases, thus leading to increased endothelial EET biosynthesis, has proved that endothelial CYP epoxygenases regulate blood pressure. In fact, these mice exhibit enhanced afferent arteriolar dilation, lower blood pressure and attenuated hypertension-induced renal injury compared to wild-type (Lee et al., 2010). These findings suggest the potential therapeutic utility of antihypertensive strategies that may increase CYP-derived EETs.

Different pharmacological strategies have been developing to increase EET availability: (i) the administration of EET analogs; (ii) the inhibition of EET catabolism by sEH inhibitors.

Epoxyeicosatrienoic acid analogs (designed to resist metabolism and improve their solubility) have been synthesized and have permit to determine EET structure function relationships and define the physiological roles of each EET regioisomer in the cardiovascular system (Sudhahar et al., 2010). Recently, it has been selected a 11,12-EET analog capable to lower blood pressure in spontaneously hypertensive rats (Sudhahar et al., 2010), supporting the use of EETs analog in the treatment of hypertension.

sEH inhibitors are efficacious antihypertensive agents in the Ang II-dependent animal model, a model of human essential hypertension (Imig et al., 2002; Bellien et al., 2011; Imig, 2012). In addition to their efficacy in lowering blood pressure, sEH inhibitors improve endothelial function and reduce hypertension-induced renal injury and cardiac hypertrophy/dysfunction. Moreover, an increasing number of studies have also shown the beneficial effects of sEH inhibitors in other cardiovascular disorders, including ischemia–reperfusion, heart failure, and atherosclerosis (Zhang et al., 2009; Bellien et al., 2011; Merabet et al., 2012).

Among numerous synthesized sEH inhibitors, AR9281 [1-(1-acetyl-piperidin-4-yl)-3-adamantan-1-yl-urea; Imig and Hammock, 2009; Anandan et al., 2011] was selected for phase 1 clinical trial. AR9281 showed a safety profile and it directly and dose-dependently inhibited blood sEH activity, in healthy subjects (Chen et al., 2012). Its efficacy in patients with hypertension and type 2 diabetes has been evaluated in phase II clinical trials, which results are not yet published, even if the trial was completed in 2009 (http://clinicaltrials.gov/show/NCT00847899).

### POTENTIAL ADVERSE EFFECTS IN TARGETING CYP-DERIVED EPOXYEICOSATRIENOIC ACID PATHWAY

Potential adverse effects of pharmacological modulation of CYP-derived EETs pathway have to be taken into account during the development of a sEH inhibitor for the treatment of cardiovascular disease.

Adverse events may occur in the pulmonary vasculature. In fact, EETs, generated in vascular smooth muscle cells, increase intracellular Ca^2+^, thus inducing vasoconstriction and increasing pulmonary artery pressure (Bellien and Joannides, 2013). It has been shown that sEH inhibitors can exacerbate hypoxic pulmonary vasoconstriction, and hypoxia induced pulmonary vascular remodeling (Pokreisz et al., 2006; Keserü et al., 2008). Increased pulmonary vasoconstriction in response to hypoxia was evidenced in Ephx2^-/-^ mice. However, 14, 15-EET is able to contrast TNF-α induced hyper-reactivity in human airway smooth muscle cells (Imig, 2012). Altogether these results suggest that sEH inhibitors are potentially associated with pulmonary vasoconstriction, but they can be beneficial in the treatment of bronchial inflammation (Imig, 2012).

Other potential unwanted cardiovascular effects may limit the therapeutic use for sEH inhibitors. In fact, even if sEH inhibitors can improve cardiac function following ischemia, they delay blood pressure recovery after cardiopulmonary resuscitation in mice and this effect was associated with higher mortality (Imig, 2012). Moreover, it has been found that EETs inhibit platelet aggregation (Heizer et al., 1991) and can hyperpolarize platelets and inactivate them by inhibiting adhesion molecule expression. They can inhibit platelet adhesion to cultured endothelial cells (Krötz et al., 2004), thus resulting in enhanced bleeding and hemorrhaging in patients taking sEH inhibitors.

sEH inhibitors can promote angiogenesis, resulting in acceleration of tumorigenesis (Michaelis et al., 2005; Fleming, 2007; Panigrahy et al., 2011; Xu et al., 2011; Imig, 2012). In fact, Panigrahy et al. (2012) have recently demonstrated that EETs have a potent stimulatory effect on primary tumor growth and tumor angiogenesis. Moreover, elevated EETs triggered extensive metastatic spread and escape from tumor dormancy in several tumor models (Panigrahy et al., 2012). In particular, in Tie2-CYP transgenic mice, a transgenic model engineered to raise endothelial EET levels, tumors that rarely metastasize, exhibit extensive metastatic spread into the majority of organs. The exogenous administration of EETs induces multiorgan metastasis and tumor dormancy escape in a variety of transplantable and genetically engineered cancer models. In contrast, the administration of EET antagonists reduced tumor growth and metastasis, prolonging mice survival (Panigrahy et al., 2012).

## CONCLUSION

Epoxyeicosatrienoic acids have many biological functions which contribute importantly to vascular physiology and to maintain cardiovascular homeostasis (Archer et al., 2003; Imig, 2012; Bellien and Joannides, 2013; Oni-Orisan et al., 2014). Thus, the increase in EET availability is emerged as a new therapeutic opportunity in the clinical management of patients at high cardiovascular risk. However, the use of EETs analogs and sEH inhibitors is limited due to the recent evidences that EETs also promote tumor growth and metastasis (**Figure 2**; Michaelis et al., 2005; Jiang et al., 2007; Panigrahy et al., 2011, 2012; Bellien and Joannides, 2013).

Appropriate clinical studies should be performed to characterize the safety profile of these novel classes of drugs before they can be considered for the treatment of cardiovascular disease in humans.

## Conflict of Interest Statement

The authors declare that the research was conducted in the absence of any commercial or financial relationships that could be construed as a potential conflict of interest.

## References

[B1] Ai D. Fu Y. Guo D. Tanaka H. Wang N. Tang C. (2007). Angiotensin II up-regulates soluble epoxide hydrolase in vascular endothelium in vitro, and in vivo. *Proc. Natl. Acad. Sci. U.S.A.* 104 9018–9023 10.1073/pnas.0703229104 17495027 PMC1885620

[B2] Alonso-Galicia M. Maier K. G. Greene A. S. Cowley A. W. Jr. Roman R. J. (2002). Role of 20-hydroxyeicosatetraenoic acid in the renal, and vasoconstrictor actions of angiotensin II. *Am. J. Physiol. Regul. Integr. Comp. Physiol.* 283 R60–R68 10.1152/ajpregu.00664.2001 12069931

[B3] Anandan S. K. Webb H. K. Chen D. Wang Y. X. Aavula B. R. Cases S. (2011). 1-(1-acetyl-piperidin-4-yl)-3-adamantan-1-yl-urea. (AR9281) as a potent, selective, and orally available soluble epoxide hydrolase inhibitor with efficacy in rodent models of hypertension and dysglycemia. *Bioorg. Med. Chem. Lett.* 21 983–988 10.1016/j.bmcl.2010.12.042 21211973 PMC3529200

[B4] Archer S. L. Gragasin F. S. Wu X. Wang S. McMurtry S. Kim D. H. (2003). Endothelium-derived hyperpolarizing factor in human internal mammary artery is 11,12. (–)epoxyeicosatrienoic acid and causes relaxation by activating smooth muscle BK(Ca) channels. *Circulation* 107 769–776 10.1161/01.CIR.0000047278.28407.C2 12578883

[B5] Bellien J. Joannides R. (2013). Epoxyeicosatrienoic acid pathway in human health, and diseases. *J. Cardiovasc. Pharmacol.* 61 188–196 10.1097/FJC.0b013e318273b007 23011468

[B6] Bellien J. Joannides R. Richard V. Thuillez C. (2011). Modulation of cytochrome derived epoxyeicosatrienoic acids pathway: a promising pharmacological approach to prevent endothelial dysfunction in cardiovascular diseases? *Pharmacol. Ther.* 131 1–17 10.1016/j.pharmthera.2011.03.015 21514320

[B7] Catella F. Lawson J. A. Fitzgerald D. J. Fitzgerald G. (1990). Endogenous biosynthesis of arachidonic acid epoxides in humans: increased formation in pregnancy induced hypertension. *Proc. Natl. Acad. Sci. U.S.A.* 87 5893–5897 10.1073/pnas.87.15.5893 2198572 PMC54435

[B8] Chen D. Whitcomb R. MacIntyre E. Tran V. Do Z. N. Sabry J. (2012). Pharmacokinetics, and pharmacodynamics of AR9281, an inhibitor of soluble epoxide hydrolase, in single-, and multiple-dose studies in healthy human subjects. *J. Clin. Pharmacol.* 52 319–328 10.1177/0091270010397049 21422238

[B9] Chen Y. Falck J. R. Manthati V. L. Jat J. L. Campbell W. B. (2011). 20-Iodo-14,15-epoxyeicosa-8(Z)-enoyl-3-azidophenylsulfonamide: photoaffinity labeling of a 14, 15-epoxyeicosatrienoic acid receptor. *Biochemistry* 50 3840–3848 10.1021/bi102070w 21469660 PMC3100183

[B10] Croft K. D. McGiff J. C. Sanchez-Mendoza A. Carroll M. A. (2000). Angiotensin II releases 20-HETE from rat renal microvessels. *Am. J. Physiol. Renal Physiol.* 279 F544–F551. 10966934 10.1152/ajprenal.2000.279.3.F544

[B11] Deng Y. Theken K. N. Lee C. R. (2010). Cytochrome P450 epoxygenases, soluble epoxide hydrolase, and the regulation of cardiovascular inflammation. *J. Mol. Cell. Cardiol.* 48 331–341 10.1016/j.yjmcc.2009.10.022 19891972 PMC2813356

[B12] Fatica R. A. Port F. K. Young E. W. (2001). Incidence trends, and mortality in end-stage renal disease attributed to renovascular disease in the United States. *Am. J. Kidney Dis.* 37 1184–1190 10.1053/ajkd.2001.24521 11382687

[B13] Fava C. Ricci M. Melander O. Minuz P. (2012). Hypertension, cardiovascular risk, and polymorphisms in genes controlling the cytochrome P450 pathway of arachidonic acid: a sex-specific relation? *Prostaglandins Other Lipid Mediat.* 98 75–85 10.1016/j.prostaglandins.2011.11.007 22173545

[B14] Fleming I. (2007). Epoxyeicosatrienoic acids, cell signaling, and angiogenesis. *Prostaglandins Other Lipid Mediat.* 82 60–67 10.1016/j.prostaglandins.2006.05.003 17164133

[B15] Fleming I. (2008). Vascular cytochrome p450 enzymes: physiology and pathophysiology. *Trends Cardiovasc. Med.* 18 20–25 10.1016/j.tcm.2007.11.002 18206805

[B16] Heizer M. L. McKinney J. S. Ellis E. F. (1991). 14,15-Epoxyeicosatrienoic acid inhibits platelet aggregation in mouse cerebral arterioles. *Stroke* 22 1389–1393 10.1161/01.STR.22.11.1389 1750047

[B17] Huang A. Sun D. Jacobson A. Carroll M. A. Falck J. R. Kaley G. (2005). Epoxyeicosatrienoic acids are released to mediate shear stress-dependent hyperpolarization of arteriolar smooth muscle. *Circ. Res.* 96 376–383 10.1161/01.RES.0000155332.17783.26 15637296 PMC4536910

[B18] Imig J. D. (2012). Epoxides, and soluble epoxide hydrolase in cardiovascular physiology. *Physiol. Rev.* 92 101–130 10.1152/physrev.00021.2011 22298653 PMC3613253

[B19] Imig J. D. Hammock B. D. (2009). Soluble epoxide hydrolase as a therapeutic target for cardiovascular diseases. *Nat. Rev. Drug Discov.* 8 794–805 10.1038/nrd2875 19794443 PMC3021468

[B20] Imig J. D. Zhao X. Capdevila J. H. Morisseau C. Hammock B. D. (2002). Soluble epoxide hydrolase inhibition lowers arterial blood pressure in angiotensin II hypertension. *Hypertension* 39 690–694 10.1161/hy0202.103788 11882632

[B21] Jiang H. McGiff J. C. Fava C. Amen G. Nesta E. Zanconato G. (2013). Maternal, and fetal epoxyeicosatrienoic acids in normotensive, and preeclamptic pregnancies. *Am. J. Hypertens.* 26 271–278 10.1093/ajh/hps011 23382413 PMC3935001

[B22] Jiang J. G. Ning Y. G. Chen C. Ma D. Liu Z. J. Yang S. (2007). Cytochrome p450 epoxygenase promotes human cancer metastasis. *Cancer Res.* 67 6665–6674 10.1158/0008-5472.CAN-06-3643 17638876

[B23] Johansson M. Herlitz H. Jensen G. Rundqvist B. Friberg P. (1999). Increased cardiovascular mortality in hypertensive patients with renal artery stenosis: relation to sympathetic activation, renal function, and treatment regimens. *J. Hypertens.* 17 1743–1750 10.1097/00004872-199917120-00012 10658941

[B24] Keserü B. Barbosa-Sicard E. Popp R. Fisslthaler B. Dietrich A. Gudermann T. (2008). Epoxyeicosatrienoic acids, and the soluble epoxide hydrolase are determinants of pulmonary artery pressure, and the acute hypoxic pulmonary vasoconstrictor response. *FASEB J.* 22 4306–4315 10.1096/fj.08-112821 18725458 PMC2614611

[B25] Kim S. Iwao H. (2000). Molecular, and cellular mechanisms of angiotensin-II mediated cardiovascular, and renal diseases. *Pharmacol. Rev.* 52 11–34. 10699153

[B26] King L. M. Gainer J. V. David G. L. Dai D. Goldstein J. A. Brown N. J. (2005). Single nucleotide polymorphisms in the CYP2J2, and CYP2C8 genes, and the risk of hypertension. *Pharmacogenet. Genomics* 15 7–13 10.1097/01213011-200501000-00002 15864120

[B27] King L. M. Ma J. Srettabunjong S. Graves J. Bradbury J. A. Li L. (2002). Cloning of CYP2J2 gene, and identification of functional polymorphisms. *Mol. Pharmacol.* 61 840–852 10.1124/mol.61.4.840 11901223

[B28] Krötz F. Riexinger T. Buerkle M. A. Nithipatikom K. Gloe T. Sohn H. Y. (2004). Membrane-potential-dependent inhibition of platelet adhesion to endothelial cells by epoxyeicosatrienoic acids. *Arterioscler. Thromb. Vasc. Biol.* 24 595–600 10.1161/01.ATV.0000116219.09040.8c 14715644

[B29] Kujal P. Čertíková Chábová V. Škaroupková P. Husková Z. Vernerová Z. Kramer H. J. (2014). Inhibition of soluble epoxide hydrolase is renoprotective in 5/6 nephrectomized Ren-2 transgenic hypertensive rats. *Clin. Exp. Pharmacol. Physiol.* 41 227–237 10.1111/1440-1681.12204 24471737 PMC4038339

[B30] Lee C. R. Imig J. D. Edin M. L. Foley J. DeGraff L. M. Bradbury J. A. (2010). Endothelial expression of human cytochrome P450 epoxygenases lowers blood pressure, and attenuates hypertension-induced renal injury in mice. *FASEB J.* 24 3770–3781 10.1096/fj.10-160119 20495177 PMC2996903

[B31] Lorthioir A. Guerrot D. Joannides R. Bellien J. (2012). Diabetic CVD–soluble epoxide hydrolase as a target. *Cardiovasc. Hematol. Agents Med. Chem.* 10 212–222 10.2174/187152512802651042 22632263

[B32] Manhiani M. Quigley J. E. Knight S. F. Tasoobshirazi S. Moore T. Brands M. W. (2009). Soluble epoxide hydrolase gene deletion attenuates renal injury, and inflammation with DOCA-salt hypertension. *Am. J. Physiol. Renal Physiol.* 297 F740–F748 10.1152/ajprenal.00098.2009 19553349 PMC2739707

[B33] Marciante K. D. Totah R. A. Heckbert S. R. Smith N. L. Lemaitre R. N. (2008). Common variation in cytochrome P450 epoxygenase genes, and the risk of incident nonfatal myocardial infarction, and ischemic stroke. *Pharmacogenet. Genomics* 18 535–543 10.1097/FPC.0b013e3282fd1287 18496133

[B34] McGiff J. C. Quilley J. (1999). 20-HETE, and the kidney: resolution of old problems, and new beginnings. *Am. J. Physiol.* 277 R607–R623. 10484476 10.1152/ajpregu.1999.277.3.R607

[B35] Merabet N. Bellien J. Glevarec E. Nicol L. Lucas D. Remy-Jouet I. (2012). Soluble epoxide hydrolase inhibition improves myocardial perfusion, and function in experimental heart failure. *J. Mol. Cell. Cardiol.* 52 660–666 10.1016/j.yjmcc.2011.11.015 22155238

[B36] Michaelis U. R. Fisslthaler B. Barbosa-Sicard E. Falck J. R. Fleming I. Busse R. (2005). Cytochrome P450 epoxygenases 2C8, and 2C9 are implicated in hypoxia-induced endothelial cell migration, and angiogenesis. *J. Cell Sci.* 118 5489–5498 10.1242/jcs.02674 16291720

[B37] Minuz P. Jiang H. Fava C. Turolo L. Tacconelli S. Ricci M. (2008). Altered release of cytochrome p450 metabolites of arachidonic acid in renovascular disease. *Hypertension.* 51 1379–1385 10.1161/HYPERTENSIONAHA.107.105395 18378855 PMC4544761

[B38] Minuz P. Patrignani P. Gaino S. Degan M. Menapace L. Tommasoli R. (2002). Increased oxidative stress, and platelet activation in patients with hypertension, and renovascular disease. *Circulation* 106 2800–2805 10.1161/01.CIR.0000039528.49161.E9 12451006

[B39] Oni-Orisan A. Alsaleh N. Lee C. R. Seubert J. M. (2014). Epoxyeicosatrienoic acids, and cardioprotection: the road to translation. *J. Mol. Cell. Cardiol.* 74C, 199–208 10.1016/j.yjmcc.2014.05.016 24893205 PMC4115045

[B40] Panigrahy D. Edin M. L. Lee C. R. Huang S. Bielenberg D. R. Butterfield C. E. (2012). Epoxyeicosanoids stimulate multiorgan metastasis, and tumor dormancy escape in mice. *J. Clin. Invest.* 122 178–191 10.1172/JCI58128 22182838 PMC3248288

[B41] Panigrahy D. Greene E. R. Pozzi A. Wang D. W. Zeldin D. C. (2011). EET signaling in cancer. *Cancer Metastasis Rev.* 30 525–540 10.1007/s10555-011-9315-y 22009066 PMC3804913

[B42] Pokreisz P. Fleming I. Kiss L. Barbosa-Sicard E. Fisslthaler B. Falck J. R. (2006). Cytochrome P450 epoxygenase gene function in hypoxic pulmonary vasoconstriction, and pulmonary vascular remodeling. *Hypertension* 47 762–770 10.1161/01.HYP.0000208299.62535.58 16505204 PMC1993904

[B43] Powell P. K. Wolf I. Jin R. Lasker J. M. (1998). Metabolism of arachidonic acid to 20-hydroxy-5,8,11, 14-eicosatetraenoic acid by P450 enzymes in human liver: involvement of CYP4F2, and CYP4A11. *J. Pharmacol. Exp. Ther.* 285 1327–1336. 9618440

[B44] Sinal C. J. Miyata M. Tohkin M. Nagata K. Bend J. R. Gonzalez F. J. (2000). Targeted disruption of soluble epoxide hydrolase reveals a role in blood pressure regulation. *J. Biol. Chem.* 275 40504–40510 10.1074/jbc.M008106200 11001943

[B45] Spector A. A. (2009). Arachidonic acid cytochrome P450 epoxygenase pathway. *J Lipid Res.* 50 S52–S6 10.1194/jlr.R800038-JLR200 18952572 PMC2674692

[B46] Spiecker M. Darius H. Hankeln T. Soufi M. Sattler A. M. Schaefer J. R. (2004). Risk of coronary artery disease associated with polymorphism of the cytochrome P450 epoxygenase CYP2J2. *Circulation* 110 2132–2136 10.1161/01.CIR.0000143832.91812.60 15466638 PMC2633457

[B47] Sudhahar V. Shaw S. Imig J. D. (2010). Epoxyeicosatrienoic acid analogs, and vascular function. *Curr. Med. Chem.* 17 1181–1190 10.2174/092986710790827843 20158473 PMC2855336

[B48] Sun D. Cuevas A. J. Gotlinger K. Hwang S. H. Hammock B. D. Schwartzman M. L. (2014). Soluble epoxide hydrolase-dependent regulation of myogenic response, and blood pressure. *Am. J. Physiol. Heart Circ. Physiol.* 306 H1146–H1153 10.1152/ajpheart.00920.2013 24561863 PMC3989753

[B49] Theken K. N. Schuck R. N. Edin M. L. Tran B. Ellis K. Bass A. (2012). Evaluation of cytochrome P450-derived eicosanoids in humans with stable atherosclerotic cardiovascular disease. *Atherosclerosis* 222 530–536 10.1016/j.atherosclerosis.2012.03.022 22503544 PMC3361525

[B50] Wang D. Dubois RN. (2012). Epoxyeicosatrienoic acids: a double-edged sword in cardiovascular diseases, and cancer. *J. Clin. Invest.* 122 19–22 10.1172/JCI61453 22182836 PMC3248310

[B51] Wang X. Ni L. Yang L. Duan Q. Chen C. Edin M. L. (2014). CYP2J2-derived epoxyeicosatrienoic acids suppress endoplasmic reticulum stress in heart failure. *Mol. Pharmacol.* 85 105–115 10.1124/mol.113.087122 24145329 PMC3868901

[B52] Weintraub N. L. Fang X. Kaduce T. L. VanRollins M. Chatterjee P. Spector A. A. (1999). Epoxide hydrolases regulate epoxyeicosatrienoic acid incorporation into coronary endothelial phospholipids. *Am. J. Physiol.* 277 H2098–H2108. 10564166 10.1152/ajpheart.1999.277.5.H2098

[B53] Xu X. Zhang X. A. Wang D. W. (2011). The roles of CYP450 epoxygenases, and metabolites, epoxyeicosatrienoic acids, in cardiovascular, and malignant diseases. *Adv. Drug Deliv. Rev.* 63 597–609 10.1016/j.addr.2011.03.006 21477627

[B54] Yu Z. Xu F. Huse L. M. Morisseau C. Draper A. J. Newman J. W. (2000). Soluble epoxide hydrolase regulates hydrolysis of vasoactive epoxyeicosatrienoic acids. *Circ. Res.* 87 992–998 10.1161/01.RES.87.11.992 11090543

[B55] Zhang L. N. Vincelette J. Cheng Y. Mehra U. Chen D. Anandan S. K. (2009). Inhibition of soluble epoxide hydrolase attenuated atherosclerosis, abdominal aortic aneurysm formation, and dyslipidemia. *Arterioscler. Thromb. Vasc. Biol.* 29 1265–1270 10.1161/ATVBAHA.109.186064 19667112

